# Linkages between share pledging, stock price risk and profitability: Evidence from the P.R. China

**DOI:** 10.1371/journal.pone.0260040

**Published:** 2021-11-18

**Authors:** Fengchao Li, Xing Zhang, Jaime Ortiz

**Affiliations:** 1 School of Finance, Renmin University of China, Beijing, P.R. China; 2 University of Houston, Houston, Texas, United States of America; University of Almeria, SPAIN

## Abstract

Share pledging has become popular as a method of loan collateral among Chinese shareholders. Our research used a sample of Chinese listed firms between 2008–2018 and produced two main findings. Firstly, we found a negative association between stock price risk and firm profitability. Our second finding was that the interaction effect of share pledging and stock price risk is greater on firm profitability than the effect of stock price risk itself. We examined the role of share pledging by modeling pooled OLS and fixed effects using share pledging behavior, controlling shareholders’ share pledging and the share pledging ratio to reinforce the robustness of our results. Furthermore, we investigated the Davis Double Play effect of share pledging to analyze how share pledging affects stock price risk. We found that higher EPS and investor expectations cannot mitigate the positive impact of share pledging on stock price risk. That is, the reduction of EPS and the deterioration of investor expectations caused by share pledging risk will not further aggravate the stock price risk, as shareholders may have taken some managerial actions to affect the transmission mechanism.

## Introduction

Turmoil in financial markets disrupts the real economy and brings uncertainty to firms. A typical example of financial risk contagion was the 2008 worldwide economic crisis which spread the risk from the financial sector to the real economy. We therefore decided to investigate how stock price risk links with corporate development. In order to guard companies against systemic financial risk, the Chinese government emphasized that the financial sector should achieve the fundamental purpose of serving the real economy. Firms usually conduct market value management to maintain the stock price and to avoid stock price volatility affecting corporate development. Conventional public opinion suggests that stock prices depend on firms’ profitability [1] which explains why the effect of stock price risk on firms’ profitability has become increasingly recognized. On the one hand, stock price risk would increase a firm’s financing costs and aggravate the financing constraints, which in turn may hinder project investment and lead to loss, which is harmful to sustainable corporate development. On the other hand, stock price risk would negatively affect a firm’s value through inhibiting corporate innovation because the firm will take less risks, including reducing innovation activities when their stock faces crash risk [2].

Share pledging has been used as loan collateral by shareholders in China despite it being viewed as unworthy in many developed countries [3]. Insider pledging in US is currently reduced due to the Institutional Shareholder Service (ISS) denouncement of pledging in 2012 [4]. Thus, China is in a pivotal place to investigate this issue. Share pledging relaxes shareholders’ personal liquidity constraints and has a lower capital cost. In 2015, it experienced a huge hit by the market meltdown in China, but then recovered strongly, peaking at 6.15 trillion RMB at the end of 2017. According to the WIND database, by 2018 almost 95.8% of Chinese A-share listed firms had shares pledged, with an accumulative pledge of 4.9 trillion RMB which accounted for about 10% of the total Chinese A-share market value. Moreover, the pledge ratio in 772 firms exceeded 50%. Shareholders who pledge their shares face an increasing risk in the event of falling stock prices. The Chinese stock market turbulence in 2018 triggered a serious share pledging risk. Regulators and firms made great efforts to mitigate the risk exposure of share pledging transmitting to the real economy, and thus formed systemic risk. A stream of studies provides empirical evidence that share pledging increase firms’ risk [5, 6]. Generally, the value of pledged shares is required to be higher than a certain level before a “margin call” is triggered as long as the stock price falls below a certain threshold [7], which would force the pledging shareholder to either add more money or pledge additional shares. Otherwise, the shares will be sold as loan collateral by the commercial bank or another financial institution that has pledged shares to lend money to the pledging shareholder. This will put further downward pressure on the stock price and, in the worst-case scenario, exacerbate the stock price crash risk. There are two competing arguments regarding the relationship between share pledging and corporate development. On the one hand, the controlling shareholder will lose control of the enterprise if the pledged shares are forced to be sold amid stock market turbulence, which may lead to further uncertainty [8]. On the other hand, the firm’s value is expected to increase because of the managerial opportunism of the pledging shareholder [9]. They will take additional actions to signal the market that the future stock price will not decrease, and a stable price will benefit corporate development [10]. Therefore, the effect of share pledging on the relationship between stock price risk and firms’ profitability remains an empirical question.

We also investigated the potential mechanisms that Davis Double Play may channel the way share pledging affects stock price risk. Davis Double Play is the double effect between market expectations and stock price fluctuations of listed firms [11]. In other words, a firm’s earnings growth gives the stock an initial boost, then investors put a higher price tag on earnings (multiple expansion), giving the stock a second boost. For example, a firm with a P/E 10 earning $1 per share is priced at $10. When the firm doubles its earnings over several years and investors show positive expectations on the stock price, the market will value the firm at a P/E 20 and the stock price will jump 4 times to $40. Conversely, decreased earnings will produce negative expectations on the stock price and the stock price will drop rapidly. Davis Double Play can explain the share pledging risk outbreak in the 2018 Chinese stock market. Market confidence collapsed when the stock price fell below the closing line for those share-pledged listed firms. Most individual investors panicked and sold the stock. It appears that if the share pledging ratio is too high, the firm’s business performance is no longer important as there will be suspicions that it’s capital chain will be broken or even go into bankruptcy. Excessive pessimism leads to an irreversible decrease in stock prices. Therefore, a dramatic move in the Chinese stock market provides a suitable background for verifying the Davis Double Play effect of share pledging.

A dramatic move in the Chinese stock market in 2018 provides a suitable theoretical framework for verifying the Davis Double Play effect of share pledging. Therefore, this study aims to offer new avenues to identify the role of share pledging in spreading risk to the real economy and can assist regulators and policy makers to better monitor the risk of share pledging and ensure the healthy and sustainable development of firms.

This paper makes two important contributions to the extant literature. Firstly, it highlights the relationship between the stock market and the real sector by providing evidence of how stock price risk affects firms’ profitability. The argument that the financial market risk will transform to the real sector in a crisis is true. However, it has been primarily discussed under a macro perspective using aggregate economic indicators, meaning that there is little research from a micro perspective, using firm-level data. Secondly, it expands the notion about the economic consequences of share pledging by examining whether share pledging affects the relationship between stock price risk and firm’s profitability. Previous studies have mostly focused on how share pledging affects firms’ behaviors, including accounting manipulations [12], real earnings management [13], auditor choice [8], corporate social responsibility [14], corporate repurchase [7], corporate risk-taking [6] and so on. Taking the influence of share pledging on stock crash risk or firm value [10, 15, 16], we investigated the amplifying role of share pledging in the relationship between stock price risk and firms’ profitability, especially the transmission mechanism of share pledging influencing stock price risk by exploring the channel of Davis Double Play effect of share pledging. To our best knowledge, there is no academic research on the Davis Double Play effect, thus our study fills this gap.

The reminder of the paper is as follows. Section 2 reviews the literature and identifies the testable hypotheses. Section 3 introduces the regression models, variables, and data. Section 4 describes the sample data and reports the empirical analysis. Section 5 presents the robustness results. Section 6 summarizes the findings and concludes.

## Literature review and research hypotheses

### Stock price risk and firm’s profitability

A number of studies have documented the interdependence between financial development and real sector output and demonstrated how financial markets drive economic growth [17]. The stock market is a mirror image of the real economy. Stock prices and macroeconomic conditions usually drop simultaneously [18]. The research by Gregoriou et al. [19] and Pan et al. [20] both supported an interaction between macroeconomic variables and stock price returns for the U.S. A higher rate of return in the stock market would encourage investment in a firm [21]. Some problems experienced by the firms may be exposed by indices in the stock market [22], such as the volatility of stock price.

Stock price risk is of concern to everyone. Excessive stock market volatility may harm the firm’s value [1, 23]. The current literature examines the relationship between the stock market and the real economy, for example, Atje et al. [24], Korajczyk [25], and Levine et al. [26] found a strong positive relationship between the stock market and economic growth. However, most of those studies were based on aggregate stock market indices [27]. We aimed to provide further evidence by analyzing the effect of stock price risk on the development ability of individual firms. There are studies on the relationship between stock risk and firm value using the indicators of Tobin’s Q. Khanna et al. [28] investigated the feedback effect of stock prices on firms’ value. They noted that firms can take advantage of a higher stock price by making more acquisitions. The stock shocks will affect the firm’s financial constraints, however, the effect of the stock risk on a firm value is inconclusive. Shin et al. [29] asserted that, from the perspective of the real option theory, firms with greater volatility would have more valuable growth opportunities.

### Share pledging

Although a growing body of literature discusses share pledging, there is a lack of research on the effect of share pledging on the relationship between stock market and corporate development. Share pledging can increase liquidity, reduce the cost of debt in real assets, stimulate the economy at the macro level and keep shareholders and corporate profits consistent [30, 31]. However, Dou et al. [32] and Ni et al. [33] both documented that share pledging would increase downside risk and reduce the value of firms. On the one hand, share pledging raises firms’ exposure to negative price shock as large stock price declines automatically trigger margin calls, which compound the downward pressure on the stock price. On the other hand, this also increases the shareholder cost which will lead firms to avoid risky projects with positive NPVs, and thus reduce the firm’s value. Dou et al. [32] also pointed out that margin calls triggered by severe price falls would exacerbate the crash risk of pledging firms. Xu et al. [34] similarly concluded that share pledging influences the stock price crash risk by producing longer suspension and greater price fluctuation. Pledged firms exhibit much higher stock price crash risk compared with non-pledged counterparts when it is the controlling shareholder that pledges the shares [35]. Yang et al. [36] found that share pledging by controlling shareholders has a significant casual negative impact on both right-tail and left-tail risk. It will exacerbate firm-risk and consequently favors lower-risk capital investments and adversely affect firms’ longer-term performance and development [5]. Shleifer et al. [37] suggested that share pledging potentially increases risk through a contingency channel. Intuitively, stock prices change often and each change increases or decreases the risk of forced liquidation of pledged shares at depressed prices.

Prior literature investigated the effect of share pledging on shareholder risk from the perspective of changing managerial incentives or contingency risk [15], and several studies attempted to provide evidence for the effect of share pledging on firms’ performance. Yeh et al. [38] documented that share pledging would reduce the value of firms through causing a severe agency problem. Hao et al. [39] reported that share pledging ratio is negatively related to firm value as it weakens the incentive effect and strengthens the entrenchment effect. However, the role of share pledging on affecting stock price volatility and the transmission mechanism is underexplored. Therefore, we further extend current findings by considering the effect of share pledging on the relationship between stock price risk and corporate development. In addition, we tested whether a Davis Double Play effect exists for share pledging and we explained the mechanism of share pledging increasing stock price risk.

### Research gap from the literature

There are many studies on the relationship between stock market and real sector, however, few examined this element from the firm-specific perspective. In particular, the influence of stock price risk on firms’ business performance is not well understood. This paper provides evidence on how stock price risk affects firms’ profitability in an emerging market such as China. Despite the fact some empirical studies confirmed the effect of share pledging on stock price crash risk, the literature is still sparse regarding how share pledging affects the stock price risk. We propose that share pledging will increase stock price risk through the transmission mechanism of Davis Double Play. There is no scholarly evidence on the Davis Double Play effect of share pledging, despite some Chinese analysts stressing its importance as being worthy of study. Thus, this study provides an empirical investigation of it.

### Hypothesis development

Stock returns, risk and profitability are significantly correlated [40]. Moreover, risk has different roles in explaining the profitability of business units [41]. Excess volatility of the stock price will harm the firm through investor sentiment [42], thus affecting financial decisions and investment. Senior management usually pays much attention to the fluctuation of stock prices as it will limit the firm’s funding ability. When a firm’s stock price falls under great volatility, investors will doubt the firm’s business and operation and choose not to invest in the firm. Hence a project cannot proceed successfully, and this will negatively affect the firm’s short-term outputs. More problematic is that R&D activities will be reduced or terminated, which in turn will affect the firm’s innovation productivity and profitability in the long run [43]. As a result, we propose the following hypothesis,

**H1:** Stock price risk is negatively related to firm’s profitability.

We considered the role of share pledging in the relationship between stock price risk and firm’s profitability. In the Chinese context, share pledging by controlling shareholders and high share pledging ratios are popular phenomena. Although share pledge is a channel to potentially relieve the financial constraints, it does not mean that funds will be invested to develop business and increase output [44]. Some pledged shares are purposed issued to acquire personal debt spend on private consumption [5, 35]. When the stock price falls to the warning line, shareholders either face the pressure of supplementary margin calls, which will affect the firm’s cash flow and business performance due to insufficient liquidity, or they will take the risk of losing control [45].

Share pledging potentially increases stock price risk regarding corporate investment and financing decisions [46]. Several studies have concluded that the stock price risk is higher in firms with a high share pledging ratio or controlling shareholders’ share-pledging. Zhou et al. [35] noted that pledged firms with higher pledging ratios were much more at risk of stock price crash. Controlling shareholders may display opportunistic behaviors [35], such as using tunneling or related party transactions to expropriate minority shareholders [45]. These actions may stifle the firm’s innovative activities and impede performance [43].

Thus, we propose the following hypotheses,

**H2:** The negative relationship effect of stock price risk on firm’s profitability is increased by having shares pledged.
**H3:** The negative relationship between stock price risk and firm’s profitability is stronger when controlling shareholders pledge their shares.
**H4:** The higher the share pledging ratio, the stronger the negative relationship between stock price risk and firm’s profitability.

Based on the assumption that share pledging affects the relationship between stock price risk and firm’s profitability, we explored the Davis Double Play effect of share pledging on stock price risk. When the stock price falls, the operating performance of the firm declines and earnings per share (EPS) will decrease. Investor expectation is an important factor affecting stock price risk [42]. Investors often have negative expectations for the firms’ experiencing huge stock shocks. According to Xiao et al. [47], market investors are more pessimistic about share pledging during challenging periods. For example, pledged shares’ mandatory liquidation triggered by the 2015 Chinese stock market crisis led to a stock price crash due to investors’ irrational behavior [34]. Therefore, two factors, EPS and negative investor expectations, will increase the stock price risk, hindering further corporate development. Research into the Davis Double Play effect provides evidence about the relationship between EPS and stock price and considers how investor expectations affect stock volatility [46, 48–50]. Alternatively, many controlling shareholders pledge their shares to obtain loans, not for the firm’s investment but for their own. In this case, for firms with a high share pledging ratio or when the controlling shareholders pledge their shares, there will be additional risks in the stock market. On this basis, we propose:

**H5:** The Davis Double Play effect on stock price risk increases with decreasing EPS and increasing negative expectations.
**H6:** Controlling shareholders’ share-pledging enhances the Davis Double Play effect of share pledging.
**H7:** The higher the share pledging ratio, the more significant the Davis Double Play effect of share pledging.

## Methodology

### Regression models

We established a panel regression Eq (1) to empirically test the relationship between stock price risk and firm’s profitability (H1). Following Hu et al. [42], we used pooled OLS to estimate coefficients. We controlled the year effect to alleviate the deviation of regression results due to the omission of variables with time trends. Moreover, to avoid potential misspecification bias in OLS estimation, we also employed a fixed effect model (FE) to produce a consistent estimator. A FE model controls individual effects and reduces deviation of the regression results caused by omission of the particularity of individual firms.


$$NetProfit_{i,t}=\beta_{0}+\beta_{1}NCSKEW_{i,t}+\sum \beta_{j}Controls_{i,t}+\varepsilon_{i,t}$$ (1)

The dependent variable, *NetProfit*, is measured as the natural logarithm of net profit, denoting the ability of firm’s profitability. The explanatory variable, *NCSKEW*, denotes a negative coefficient of skewness, measuring stock price risk. The bigger the *NCSKEW*, the higher the stock price risk. We measured *NCSKEW* by following Chen et al. [51], Kim et al. [52], Zhou et al. [53], and Xie et al. [54]. Firstly, we used the following expanded market model regression to estimate firm-specific weekly returns for firm *i* and year *t*:

$$r_{it}=\alpha_{i}+\beta_{1}r_{m,t-2}+\beta_{2}r_{m,t-1}+\beta_{3}r_{m,t}+\beta_{4}r_{m,t+1}+\beta_{5}r_{m,t+2}+\varepsilon_{i,t}$$

where *r*_*it*_ is the return of stock *i* during week *t* and *r*_*m*,*k*_ is the return of market index during week *k*. Thus, we got,

$$w_{i,t}=ln(1+\varepsilon_{i,t})$$

where *w*_*i*,*t*_ is weekly return for firm *i* during week *t*.

Then, *NCSKEW* is calculated by

$${NSCKEW}_{i,t}=-[n{\left(n-1\right)}^{\frac{3}{2}}\sum w_{i,t}^{3}]/[(n-1)(n-2)/{\left(\sum w_{i,t}^{2}\right)}^{3/2}]$$

where *n* is the number of total trading weeks in year *t* for stock *i*.

We used *DUVOL*, denoting down and up volatility, in robustness checks. Firstly, we separated all the weeks to “down” weeks (returns below the annual average) and “up” weeks (returns above the annual average). Then we separately calculated the standard deviation for two subsamples. Finally, we got,

$${DUVOL}_{i,t}=log\left\{[(n_{u}-1)\sum_{down}w_{i,t}^{2}\right]/\left[(n_{d}-1)\sum_{up}w_{i,t}^{2}]\right\}$$

where *n*_*u*_(*n*_*d*_) are the number of up (down) weeks during year *t*, respectively.

In addition, according to Jiraporn et al. [55], Li et al. [10], and Anderson et al. [15], we controlled the variables concerning corporate characteristics and governance: stock returns (*Return*), stock volatility (*Sigma*), firm size (*Size*), fixed asset investment (*FixSize*), financial leverage (*Leverage*), return on assets (*Roa*), operating revenue (*Ato*), net cash flow (*Ocf*), shareholder structure (*Cr5*, *Z_index*), and board structure (*GMDual*, *BoardSize*, *IndepDir*). The variable definitions are summarized in Table 1.

**Table 1:** Variables and definitions.

Variables		Definition
Dependent variables	*NetProfit*	Natural logarithm of net profit
	*Profit*	Net profit divided by total assets
Independent variables	*NCSKEW*	Negative coefficient of skewness, measuring stock price risk, calculated by referring to Chen et al. [51], Kim et al. [52], Zhou et al. [53], and Xie et al. [54].
	*DUVOL*	Down and up volatility, measuring stock price risk, calculated by referring Chen et al. [51], Kim et al. [52], Zhou et al. [53], and Xie et al. [54].
Moderating variables	*Pledge*	1 if the company takes share pledging behavior in the year, and 0 otherwise
	*PleCont*	1 if controlling shareholders pledge the shares, and 0 otherwise
	*PleRatio*	Share pledge ratio out of total shares
	*EPS*	Earnings per share, net profit divided by the number of common shares
	*IE*	Investor negative expectations ratio. Investors’ messages in the largest and most representative stock message board, *Eastmoney*.*com* in P.R. China, are classified into three types of expectations—positive, negative, and neutral. Investor negative expectations ratio denotes the number of negative messages divided by total messages.
Control variables	*Return*	Average of stock price returns
	*Sigma*	Standard deviation of stock price returns
	*Size*	Natural logarithm of total assets
	*FixSize*	Natural logarithm of net fixed assets
	*Leverage*	Total liabilities divided by total assets
	*Roa*	Return on assets
	*Ato*	Operating revenues divided by total assets
	*Ocf*	Natural logarithm of the net cash flow from operating activities
	*Cr5*	Shareholding ratio of the top five shareholders
	*Z_index*	Largest shareholder’s shareholding ratio divided by the second shareholder’s shareholding ratio
	*GMDual*	1 if the general manager is also the chair of the board of directors, and 0 otherwise
	*BoardSize*	Natural logarithm of the number of directors of board
	*IndepDir*	Proportion of independent directors on the board

We constructed Eq (2) to investigate the effect of share pledging on the relationship between stock price risk and firm’s profitability (H2) by introducing a share pledging variable, *Pledge*, and the interaction of stock price risk and share pledging, NCSKEW * Pledge, on the basis of Eq (1). We further tested the H3 and H4 by replacing the share pledging variable (*Pledge*) with controlling shareholders’ share-pledging variable (*PleCont*) and share pledging ratio variable (*PleRatio*) and then made a regression using Eq (2) respectively. As in Eq (1), we adopted the pooled OLS and the FE model for regression, with the year effect controlled.

$$NetProfit_{i,t}=\beta_{0}+\beta_{1}NCSKEW_{i,t}+\beta_{2}Pledge_{i,t}+\beta_{3}NCSKEW_{i,t}*Pledge_{i,t}+\sum \beta_{j}Controls_{i,t}+\varepsilon_{i,t}$$ (2)

where *Pledge* equals to one if the company takes share pledging behavior and zero otherwise. We separately replaced *Pledge* with *PleCont* and *PleRatio* to further assess the impact of controlling shareholders’ share pledging and share pledging ratio. *PleCont* equals to one if controlling shareholders pledge the shares and zero otherwise, and *PleRatio* is the ratio of share pledging.

In order to test the Davis Double Play effect of share pledging (H5), we constructed Eq (3). We separately replaced *Pledge* with *PleCont* and *PleRatio* to test H6 and H7. We also adopted the pooled OLS and the FE model for regression, with the year effect controlled.

$$NCSKEW_{i,t}=\beta_{0}+\beta_{1}Pledge_{i,t}+\beta_{2}EPS_{i,t}*Pledge_{i,t}+\beta_{3}IE_{i,t}*Pledge_{i,t}+\sum \beta_{j}Controls_{i,t}+\varepsilon_{i,t}$$ (3)

where *EPS* denotes the earnings per share and *IE* denotes the investor negative expectations, defined in Table 1.

### Sample selection and data

Our initial sample comprised all firms listed on the Shanghai and Shenzhen Stock Exchanges between 2008 and 2018. Financial and corporate governance data were collected from the Chinese Stock Market and Accounting Research database (CSMAR). The data on share pledging, whether firms pledged or not and the share pledging ratio, were also obtained from the CSMAR, while controlling shareholders’ share pledging data were sourced from the WIND database. The data on investor expectations were collected from the Chinese Research Data Services (CNRDS). We removed the following data, (1) listed companies on the Science and Technology Board; (2) listed companies in the financial industry; (3) special treatment (ST and *ST) listed companies; (4) samples with annual trading weeks less than 30; (5) observations in the IPO year and (6) missing values for all variables. The final sample consisted of 20,434 firm-year observations comprising 3,012 listed firms.

## Empirical results

### Descriptive statistics

Table 2 shows the descriptive statistics of share pledging. Panel A includes information about the number and ratio of share pledging. Overall, 42.8% of the firm-year observations pledged the shares. During 2008–2018, the number of pledged firms increased year by year, from 21.2% in 2008 to 58.1% in 2018, and reached a maximum of 59.7% in 2017. More than 70% of the firm-year observations pledged the shares by controlling shareholders during the sample period. The average share pledging ratio is 19.5%, and the highest is 24.1% in 2014, which represents a decrease in recent years. Panel B counts information on the share pledging ratio in different ranges. Most of firms have the share pledging ratio between 0 and 40%, some close to 100% or even more than 100%. The data confirms that having shares pledged, controlling shareholders’ share pledging, or a high share pledging ratio are three popular financing behaviors among the Chinese listed companies.

**Table 2:** Summary statistics.

Panel A: Summary statistics on share pledging							
Year	Pledge		No Pledge N	Total N	Pledge Controlling		Pledge Ratio %
	N	%			N	%	
2008	221	21.15	824	1045	160	72.40	19.65
2009	262	22.92	881	1143	195	74.43	23.02
2010	266	21.63	964	1230	200	75.19	20.30
2011	429	27.39	1137	1566	308	71.79	20.31
2012	542	29.34	1305	1847	387	71.40	19.86
2013	744	37.05	1264	2008	539	72.45	22.00
2014	833	41.92	1154	1987	642	77.07	24.09
2015	1022	51.20	974	1996	804	78.67	21.12
2016	1264	56.68	966	2230	1015	80.30	22.49
2017	1465	59.65	991	2456	1155	78.84	17.92
2018	1700	58.10	1226	2926	1329	78.18	13.45
Total	8748	42.81	11686	20434	6734	76.98	19.53
Panel B: Range of share pledging ratio							
Year	0~20%	20~40%	40~60%	60~80%	80~100%	>100%	Total
2008	142	53	19	7	0	0	221
2009	138	85	20	13	5	1	262
2010	161	77	17	6	5	0	266
2011	264	117	30	12	4	2	429
2012	328	161	30	15	6	2	542
2013	431	199	83	20	5	6	744
2014	448	244	82	31	17	11	833
2015	608	284	82	27	11	10	1022
2016	693	376	139	38	10	8	1264
2017	945	403	105	8	2	2	1465
2018	1306	350	35	8	1	0	1700
Total	5464	2349	642	185	66	42	8748

Table 3 reports the correlation coefficient between all variables. The negative correlation coefficient between *NetProfit* and *NCSKEW* shows that the increase in stock price risk reduce firm’s profitability. The positive correlation coefficient between *NCSKEW* and share pledging variables *(Pledge*, *PleCont* and *PleRatio*) shows that pledging firms, controlling shareholders’ share pledging or a high ratio of share pledging are exposed to higher stock price risk. Correlation coefficients between variables are almost consistently below 0.5 indicating a low risk of multicollinearity.

**Table 3:** Correlation coefficient test.

	*NetProfit*	*Profit*	*NCSKEW*	*DUVOL*	*Pledge*	*PleCont*	*PleRatio*
*NetProfit*	1.000						
*Profit*	0.980***	1.000					
*NCSKEW*	-0.020***	-0.022***	1.000				
*DUVOL*	-0.022***	-0.026***	0.873***	1.000			
*Pledge*	-0.060***	-0.064***	0.063***	0.061***	1.000		
*PleCont*	-0.045***	-0.047***	0.056***	0.053***	0.810***	1.000	
*PleRatio*	-0.040***	-0.042***	0.030***	0.031***	0.650***	0.621***	1.000

Notes

* p<0.1

** p<0.05

*** p<0.01.

### Basic analysis

#### The relationship between stock price risk and firm’s profitability

Table 4 presents the results after using the pooled OLS and the FE model to estimate Eq (1) respectively. Column (1) reports the results from the pooled OLS regression. The estimated coefficient of *NCSKEW* is negative and significant in predicting firm’s profitability (*NetProfit*) (p < .01). The estimated results from the FE model are reported in Column (3). We found a significantly negative association between *NCSKEW* and *NetProfit* (p < .01). These results verify hypothesis H1 by indicating that stock price risk is negatively associated with firm’s profitability.

**Table 4:** Basic and further analysis.

	Basic analysis			
	Pooled OLS		FE	
	(1)	(2)	(3)	(4)
	*NetProfit*	*NetProfit*	*NetProfit*	*NetProfit*
*NCSKEW*	-0.285***	-0.223***	-0.244***	-0.158**
	(-5.99)	(-3.69)	(-4.96)	(-2.55)
*Pledge*		-0.0690		0.00361
		(-1.10)		(0.04)
NCSKEW * Pledge		-0.138		-0.203**
		(-1.49)		(-2.11)
*Constant*	-6.905***	-6.868***	-11.25***	-11.23***
	(-9.29)	(-9.22)	(-6.73)	(-6.72)
*Year F*.*E*	Yes	Yes	Yes	Yes
*Firm F*.*E*	No	No	Yes	Yes
*N*	20434	20434	20434	20434
*adj*.*R*^*2*^	0.395	0.395	0.354	0.354
	Further analysis			
	Pooled OLS		FE	
	(5)	(6)	(7)	(8)
	*NetProfit*	*NetProfit*	*NetProfit*	*NetProfit*
*NCSKEW*	-0.249***	-0.294***	-0.177***	-0.194***
	(-4.41)	(-5.28)	(-3.09)	(-3.40)
*PleCont*	0.0225		0.0909	
	(0.34)		(0.97)	
NCSKEW * PleCont	-0.113		-0.205**	
	(-1.14)		(-2.02)	
*PleRatio*		0.309		0.836**
		(1.20)		(2.31)
NCSKEW * PleRatio		0.0774		-0.680*
		(0.20)		(-1.69)
*Constant*	-6.956***	-6.985***	-11.17***	-11.15***
	(-9.34)	(-9.38)	(-6.67)	(-6.65)
*Year F*.*E*	Yes	Yes	Yes	Yes
*Firm F*.*E*	No	No	Yes	Yes
*N*	20434	20434	20434	20434
*adj*.*R*^*2*^	0.395	0.395	0.354	0.354

Notes: (1) the estimators of control variables are not reported for saving space. (2) t statistics in parentheses. (3)

* p<0.1

** p<0.05

*** p<0.01.

#### The effect of share pledging

We added two variables, *Pledge* and the interactive variable NCSKEW * Pledge, to estimate the role of share pledging on the relationship between stock price risk and corporate development. We also used the pooled OLS and the FE model to estimate Eq (2) respectively. Column (2) summarizes the results from the pooled OLS regression. The estimated coefficient between *NCSKEW* and *NetProfit* is significantly negative at -0.223 (p < .01). The coefficient between NCSKEW * Pledge and *NetProfit* is negative but not significant (p > .1). Therefore, the results reject the hypothesis H2. The results from the FE model presented in Column (4). We found a significantly negative association between *NCSKEW* and *NetProfit* (p < .01) and a significantly negative association between NCSKEW * Pledge and *NetProfit* (p < .05). These results verify hypothesis H2 by indicating that having shares pledged increases the negative association between *NCSKEW* and *NetProfit*. In addition, the regression coefficients show that the effect of the interaction of share pledging and stock price risk (NCSKEW * Pledge) is significantly greater than the effect of stock price risk itself (*NCSKEW*) on firm’s profitability (0.203 > 0.158).

### Further analysis

#### The effect of controlling shareholders’ share pledging

We aimed to estimate whether other share pledging phenomena have similar effects. First, we re-estimated Eq (2) by replacing the share pledging variable with controlling shareholders’ share-pledging (*PleCont*). The results are shown in Table 4. Columns (5) and (7) present a significantly negative association between *NCSKEW* and *NetProfit* (p < .01) and a negative association between NCSKEW * PleCont and *NetProfit*. However, the significance of the association between NCSKEW * PleCont and *NetProfit* is different. It is significant in the FE estimation (p < .05) but not significant in the pooled OLS estimation (p > .1). These results are the same as the effect of the share pledging analysis that we reported. The results estimated from the FE model verify hypothesis H3 which states that controlling shareholders having shares pledged increases the negative association between *NCSKEW* and *NetProfit*, and the effect of the interaction of shareholders’ share-pledging. Furthermore, that stock price risk (NCSKEW * Plecont) is significantly greater than the effect of stock price risk itself (*NCSKEW*) on firm’s profitability (0.205 > 0.177).

#### The effect of share pledging ratio

We re-estimated Eq (2) by replacing the share pledging variable with share pledging ratio (*PleRatio*). The pooled OLS and the FE model regression results are shown in Table 4. Columns (6) and (8) underscore a significantly negative association between *NCSKEW* and *NetProfit* (p < .01). However, the relationship between NCSKEW * PleRatio and *NetProfit* is different. It is significantly negative in the FE regression (p < .1), but positive and non-significant in the pooled OLS estimation (p > .1). The FE estimation results verify hypothesis H4 that a higher share pledging ratio increases the negative association between *NCSKEW* and *NetProfit*. In addition, it confirms that the effect of the interaction between share pledging ratio and stock price risk (NCSKEW * Pleratio) is significantly greater than the effect of stock price risk itself (*NCSKEW*) on firms’s profitability (0.68 > 0.194).

In summary, the regressions described above prove hypotheses H2, H3 and H4. Share pledging, including having shares pledged by controlling shareholders and high share pledging ratios, can increase the negative association between stock price risk and firm’s profitability. In addition, the effect of share pledging is greater than the effect of stock price risk itself on firm’s profitability. This means that pledging firms need to pay more attention to the risk of share pledging while share pledging meets the firm’s financing demand.

### Evidence on Davis double play effect of share pledging

#### Evidence of share pledging

In this section, we analyzed whether the Davis Double Play effect of share pledging exists. On the one hand, we estimated the relationship between share pledging and stock price risk; on the other hand, we find out the effect of EPS and investor expectations on the relationship between share pledging and stock price risk. Table 5 presents the regression results for Eq (3). Columns (1) and (2) show a significantly positive association between *Pledge* and *NCSKEW* (p < .01). This means pledging firms are exposed to higher stock price risk. Obviously, we also need to consider the impact of interaction terms. The negative and significant estimated coefficient between IE * Pledge and *NCSKEW* (p < .01) implies that the lower investor negative expectations (higher investor expectations) increase the positive association between share pledging and stock price risk. The positive coefficient between EPS * Pledge and *NCSKEW* indicate that the higher EPS increases the positive association between share pledging and stock price risk. Although the results reject hypothesis H5, further analysis is required because of the non-significant coefficient of EPS * Pledge (p > .1).

**Table 5:** Davis double play effect of share pledging.

	Share pledging									
	Full sample			Sub: state-owned				Sub: non state-owned		
	OLS	FE		OLS		FE		OLS		FE
	(1)	(2)		(3)		(4)		(5)		(6)
	*NCSKEW*	*NCSKEW*		*NCSKEW*		*NCSKEW*		*NCSKEW*		*NCSKEW*
*Pledge*	0.228***	0.156***		0.412***		0.377***		0.170***		0.146**
	(5.07)	(2.99)		(3.99)		(3.38)		(3.16)		(2.28)
EPS * Pledge	0.0343	0.0272		-0.0128		-0.0773		0.0708***		0.0563*
	(1.55)	(0.97)		(-0.27)		(-1.38)		(2.71)		(1.66)
IE * Pledge	-0.760***	-0.538***		-1.360***		-1.284***		-0.704***		-0.544**
	(-4.37)	(-2.72)		(-3.38)		(-3.04)		(-3.42)		(-2.25)
*Constant*	-0.131	-0.297		-0.0950		0.266		-0.329**		-0.723**
	(-1.35)	(-1.36)		(-0.70)		(0.81)		(-2.24)		(-2.48)
*Year F*.*E*	Yes	Yes		Yes		Yes		Yes		Yes
*Firm F*.*E*	No	Yes		No		Yes		No		Yes
*N*	20434	20434		8138		8138		12296		12296
*adj*.*R*^*2*^	0.0680	0.0640		0.0750		0.0720		0.0600		0.0620
			Controlling shareholder				Share pledging ratio			
			Full sample				Sub: pledged			
			OLS		FE		OLS		FE	
			(7)		(8)		(9)		(10)	
			*NCSKEW*		*NCSKEW*		*NCSKEW*		*NCSKEW*	
*PleCont*			0.181***		0.123**					
			(3.75)		(2.21)					
EPS * PleCont			0.0425*		0.0315					
			(1.74)		(1.00)					
IE * PleCont			-0.582***		-0.469**					
			(-3.08)		(-2.18)					
*PleRatio*							0.789***		0.686**	
							(3.46)		(2.40)	
EPS * PleRatio							0.258**		-0.0155	
							(2.07)		(-0.09)	
IE * PleRatio							-3.151***		-2.927***	
							(-3.49)		(-2.68)	
*Constant*			-0.117		-0.312		-0.160		-0.540	
			(-1.21)		(-1.44)		(-0.93)		(-1.44)	
*Year F*.*E*			Yes		Yes		Yes		Yes	
*Firm F*.*E*			No		Yes		No		Yes	
*N*			20434		20434		8748		8748	
*adj*.*R*^*2*^			0.0670		0.0640		0.0570		0.0580	

Notes: (1) the estimators of control variables are not reported for saving space. (2) t statistics in parentheses. (3)

* p<0.1

** p<0.05

*** p<0.01.

We considered two subsamples, state-owned and non-state-owned enterprises, to analyze the difference of enterprise nature in the Davis Double Play effect of share pledging. For state-owned enterprises, in Columns (3) and (4), the signs and significance of the coefficients of *Pledge* and IE * Pledge are the same as the full sample (p < .01). The coefficient of EPS * Pledge turns to negative, but still not significant (p > .1). These results cannot help us verify or reject hypothesis H5, either. For non-state-owned enterprises, in Columns (5) and (6), the signs and significance of the coefficients of *Pledge* and IE * Pledge are almost the same as the full sample (p < .01 in OLS and p < .05 in FE). Meaningfully, the coefficient of EPS * Pledge is positive and significant (p < .01 in OLS and p < .1 in FE). These results reject hypothesis H5 in that lower EPS and/or the lower investor expectations alleviate the positive impact of having shares pledged on stock price risk. In other words, the Davis Double Play effect of share pledging does not exist.

#### Evidence of controlling shareholders’ share pledging and share pledging ratio

According to Cheng [56], the Davis Double Play effect of share pledging mainly exists in firms with shares pledged by controlling shareholders and high share pledging ratios. Hence, we further estimated whether controlling shareholders’ share pledging and share pledging ratio have similar results with having shares pledged. We noticed that 80% of the pledging firms in the sample belong to controlling shareholders’ share pledging. Thus, we used the full sample to investigate the role of controlling shareholders in the Davis Double Play effect of share pledging. However, we selected the subsample of pledging firms to better observe whether there is any evidence that the higher the ratio, the more significant the Davis Double Play effect of share pledging.

We re-estimated Eq (3) by replacing the share pledging variable with *PleCont* and *PleRatio* respectively. The regression results are summarized in Table 6. In Columns (7) and (9), the pooled OLS regression results present a significantly positive coefficient of *PleCont* / *PleRatio* (p < .01), a significantly positive coefficient of EPS * PleCont / EPS * PleRatio (p < .1 / p < .05) and a significantly negative coefficient of IE * PleCont / IE * PleRatio (p < .01). In Columns (8) and (10), the FE regression results present a significantly positive coefficient of *PleCont* / *PleRatio* (p < .05) and a significantly negative coefficient of IE * PleCont / IE * PleRatio (p < .05 / p < .01). However, the coefficient of EPS * PleCont / EPS * PleRatio is not significant (p > .1). The results of the pooled OLS regression reject hypotheses H6 and H7, which further verifies that the Davis Double Play effect of share pledging does not exist.

**Table 6:** Robustness checks: Alternative proxies.

	Alternative method			Alternative proxy: *Atp*					
	SYS-GMM			Pooled OLS			FE		
	(1)	(2)		(3)	(4)		(5)		(6)
	*NetProfit*	*NetProfit*		*Atp*	*Atp*		*Atp*		*Atp*
*NCSKEW*	-.0634**	-.0543***		-0.0947***	-0.117***		-0.0160***		-0.0541**
	(-1.79)	(-2.88)		(-4.49)	(-2.94)		(-2.61)		(-2.44)
*Pledge*		-0.894**			-0.0986**				0.0302
		(-2.15)			(-2.07)				(0.59)
*NCSKEW*		-0.0920*			-0.0588*				-0.0874**
* Pledge		(-1.91)			(1.85)				(1.97)
*L*.*NetProfit*	-.17894***	-.1990***							
	(-9.01)	(-8.99)							
*Constant*	7.6555	6.5101		-1.396	-1.251		-1.790		-1.781
	(0.34)	(0.40)		(-1.27)	(-1.11)		(-0.73)		(-0.73)
*AR(2)*	-0.49	-0.55							
	(0.625)	(0.583)							
*Hansen*	113.52	220.53							
	(0.121)	(0.330)							
*Year F*.*E*				Yes	Yes		Yes		Yes
*Firm F*.*E*				No	No		Yes		Yes
*N*				20434	20434		20434		20434
*adj*.*R*^*2*^				0.888	0.888		0.880		0.880
	Alternative proxy: *DUVOL*								
	Pooled OLS					FE			
	(7)		(8)			(9)		(10)	
	*NetProfit*		*NetProfit*			*NetProfit*		*NetProfit*	
*DUVOL*	-0.318***		-0.238***			-0.257***		-0.156*	
	(-4.79)		(-2.83)			(-3.86)		(-1.85)	
*Pledge*			-0.0699					0.0129	
			(-1.13)					(0.14)	
*DUVOL*			-0.178					-0.241*	
* Pledge			(-1.37)					(-1.79)	
*Constant*	-6.982***		-6.941***			-11.31***		-11.28***	
	(-9.39)		(-9.32)			(-6.76)		(-6.74)	
*Year F*.*E*	Yes		Yes			Yes		Yes	
*Firm F*.*E*	No		No			Yes		Yes	
*N*	20434		20434			20434		20434	
*adj*.*R*^*2*^	0.394		0.394			0.353		0.354	

Notes: (1) the estimators of control variables are not reported for saving space. (2) t statistics in parentheses. (3)

* p<0.1

** p<0.05

*** p<0.01.

Overall, we found consistent empirical evidence to reject the hypothesis of the Davis Double Play effect of share pledging. This may be because firms or shareholders have incentives to keep the stock price stable to reduce control right transferring risks triggered by the margin call [43]. They will take certain actions, such as colluding with analysts [42], accounting manipulations [12] and real earnings management [13] to prevent the exposure of potential share pledging risk due to the existence of share pledging, especially controlling shareholder’s share pledging and high pledging ratios. It can be concluded that share pledging will increase stock price risk. However, due to the risk management measures adopted by firms or controlling shareholders who face the share pledging risk, the reduction of EPS and the deterioration of investor expectations caused by share pledging risk will not further aggravate the stock price risk. Thus, our findings cannot prove that EPS and investor expectations help mitigate the impact of share pledging on stock price risk. We conclude that the Davis Double Play effect of share pledging during the sample period does not exist. Future research should focus on whether the Davis Double Play effect of share pledging will lead to an increase in stock price risk, and what measures firms or controlling shareholders have taken to control the share pledging risk.

## Robustness checks

To avoid the non-randomness of the empirical results and enhance their reliability and stability, we conducted robustness tests based on alternative econometric approaches and different proxies for corporate development and stock price risk, subsamples of state-owned and non-state-owned firms, as well as the hysteresis effect of share pledging.

### Alternative econometric methodologies

Endogeneity is prevalent across corporate governance studies. Following Aljughaiman et al. [57], we used a dynamic panel system generalized moment estimation method (SYS-GMM) to solve the potential endogeneity issues that existed in our studies. SYM-GMM not only controls the endogenous correlation between the first-order lag term of the explained variable and the error term, but also controls a potential endogenous correlation between the explanatory variable and the control variable and the error term [58]. We constructed the following regression model:

$${NetProfit}_{i,t}=\beta_{0}+\beta^{\prime }{NetProfit}_{i,t-1}+\beta_{1}{NCSKEW}_{i,t}+\sum \beta_{j}{Controls}_{i,t}+\varepsilon_{i.t}$$

$${NetProfit}_{i,t}=\beta_{0}+\beta^{\prime }{NetProfit}_{i,t-1}+\beta_{1}{NCSKEW}_{i,t}+\beta_{2}{Pledge}_{i,t}+\beta_{3}{NCSKEW}_{i,t}*{Pledge}_{i,t}+\sum \beta_{j}{Controls}_{i,t}+\varepsilon_{i.t}$$

where, *NetProfit*_*i*,*t*−1_ is the first-order lag term of *NetProfit*_*i*,*t*_.

In accordance with Blundell et al. [58] and Guidara et al. [59], we chose the lagged values and first-order difference of the *NetProfit*, and the first-order difference of *NCSKEW*, *Pledge* as instrumental variables in the SYS-GMM estimation. Furthermore, we used the financial shock of 2015 (*shock*) as an exogenous instrumental variable referring to Xu et al. [34]. *Shock* is the time dummy variable that distinguishes pre-crisis and post-crisis. The value equals to 1 if the sample is after 2015 and 0 otherwise. Following Zhang et al. [60], the Chinese political cycle, *pc*, was used as another exogenous instrumental variable. *pc* = 1 in the year holding the National Congress of the Communist Party of China, *pc* = 0, otherwise. Columns (1)- (2) in Table 6 show the results estimated by the SYS-GMM. The statistics of the AR (2) test and the Sargan test confirmed that the selected instrumental variables were valid. The estimated results by SYS-GMM reinforced the fact that stock price risk deteriorates firms’ profitability and share pledging amplifies such effects, consistent with previous conclusions.

### Alternative proxies of variables

We used net profits divided by total asset, *Atp*, as a proxy for firms’ profitability, which is a common practice in corporate finance literature, and estimated Eq (1) and (2) by pooled OLS and FE model. The empirical results are summarized in Columns (3–6) of Table 6. Columns (3) and (5) show the negative effect of stock price risk on firm’s profitability as *NCSKEW* has a significantly negative coefficient to *Atp*. The estimated coefficients of NCSKEW * Pledge in pooled OLS and FE model, shown in Columns (4) and (6), are -0.0588 and -0.0874, respectively, which means share pledging amplified the effect of stock price risk on firms’ profitability. These results are all consistent with previous findings.

Similarly, we carried out another robustness check by using down and up volatility (*DUVOL*) as a proxy of stock price risk. These results are summarized in Columns (7–10). Columns (7) and (9) show that the relationships between stock price risk and firm’s profitability as the coefficients of *DUVOL* were both significantly negative. Moreover, DUVOL * Pledge has a significant coefficient of -0.241, indicating that share pledging played an amplifying role in stock price risk hindering corporate development, supporting the robustness of previous conclusions.

### Subsamples and hysteresis effect

We further tested the relationship between stock price risk and firm’s profitability for state-owned and non-state-owned enterprises. The estimation results are summarized in Columns (1–8) of Table 7 and indicate that the negative effect of stock price risk on firms’ profitability concluded in the full sample is also found in the subsamples.

**Table 7:** Robustness checks: Subsamples and hysteresis effect.

	State-owned			
	Pooled OLS		FE	
	(1)	(2)	(3)	(4)
*NCSKEW*	-0.220***	-0.156*	-0.151*	-0.109
	(-2.79)	(-1.91)	(-1.90)	(-1.34)
*Pledge*		-0.318**		-0.0493
		(-2.44)		(-0.30)
NCSKEW * Pledge		-0.341*		-0.254
		(-1.75)		(-1.30)
*Constant*	-6.233***	-6.196***	-13.06***	-13.06***
	(-5.72)	(-5.93)	(-5.41)	(-5.93)
*Year F*.*E*	Yes	Yes	Yes	Yes
*Firm F*.*E*	No	No	Yes	Yes
*N*	8138	8138	8138	8138
*adj*.*R*^*2*^	0.376	0.376	0.330	0.249
	Non state-owned			
	Pooled OLS		FE	
	(5)	(6)	(7)	(8)
*NCSKEW*	-0.326***	-0.270***	-0.324***	-0.178*
	(-5.50)	(-3.08)	(-5.27)	(-1.95)
*Pledge*		0.0917		0.0702
		(1.25)		(0.72)
NCSKEW * Pledge		-0.0976		-0.195*
		(-0.88)		(-1.68)
*Constant*	-7.477***	-8.202***	-11.81***	-9.555***
	(-7.14)	(-8.87)	(-5.50)	(-5.89)
*Year F*.*E*	Yes	Yes	Yes	Yes
*Firm F*.*E*	No	No	Yes	Yes
*N*	12296	12296	12296	12296
*adj*.*R*^*2*^	0.411	0.406	0.379	0.239
		Hysteresis effect		
		Pooled OLS		FE
		(9)		(10)
*NCSKEW*		-0.163**		-0.122*
		(-2.50)		(-1.85)
*L*.*Pledge*		-0.143**		-0.0885
		(-2.00)		(-0.90)
*NCSKEW* * *L*.*Pledge*		-0.238**		-0.251**
		(-2.29)		(-2.43)
*Constant*		-7.681***		-13.49***
		(-10.12)		(-7.11)
*Year F*.*E*		Yes		Yes
*Firm F*.*E*		No		Yes
*N*		16957		16957
*adj*.*R*^*2*^		0.397		0.345

Notes: (1) the estimators of control variables are not reported for saving space. (2) t statistics in parentheses. (3)

* p<0.1

** p<0.05

*** p<0.01.

Finally, we used *L*.*Pledge*, the lag of *Pledge*, to examine whether there exists a hysteresis effect of share pledging. The coefficients of *NCSKEW* and NCSKEW * L.*Pledge* estimated by pooled OLS and FE model were both significantly negative, indicating share pledging in last period will affect corporate development in current period, which is also consistent with the effect of share pledging that was concluded in the above context.

## Conclusion

This paper examined the relationship between stock price risk and firm’s profitability, and the effect of share pledging on this relationship, as well as the Davis Double Play effect of share pledging in the Chinese stock market between 2008 and 2018. Firstly, there is a significant and negative association between stock price risk and firms’ profitability. This empirical evidence confirmed that a higher stock price risk slows down or damages the development of a firm. Secondly, we uncovered that share pledging can increase the negative association between stock price risk and firm’s profitability. In addition, we found that the effect of share pledging is greater than the effect of stock price risk itself on firm’s profitability. Therefore, pledging firms need to pay close attention to the risk of share pledging while share pledging meets the firm’s financing demand. Lastly, we concluded that the Davis Double Play effect of share pledging did not exist during the observation period. However, we still estimated the association between share pledging and stock price risk, which is positive. As firms or controlling shareholders having shares pledged realize the seriousness of share pledging risk, they will take some actions to prevent the exposure of share pledging risk. The effect may be reflected in the stock price risk at some point in the future.

Limitations of relevant data and information prevented further analysis. Future research should focus on the transmission mechanism of share pledging risk and the hysteresis of the Davis Double Play effect of share pledging.

## Supporting information

S1 Appendix Data set. (XLSX)
